# Supplementary material for: Glucose Hydrogenolysis into 1,2-Propanediol Using a Pt/deAl@Mg(OH)2 Catalyst: Expanding the Application of a Core–Shell Structured Catalyst
Source: Nanomaterials (Basel). 2022 Oct 26;12(21):3771. doi: 10.3390/nano12213771 (PMC9657227; doi:10.3390/nano12213771)
Supplement: Supplementary file 1 [file nanomaterials-12-03771-s001.zip › nanomaterials-1970377-supplementary.pdf]

nanomaterials

Supplementary Materials

Glucose Hydrogenolysis into 1,2-Propanediol Using a Pt/deAl@Mg(OH)₂ Catalyst: Expanding the Application of a Core–Shell Structured Catalyst

Shizhuo Wang, Jikang Jiang, Minyan Gu, Yuanbo Song, Jiang Zhao *, Zheng Shen *, Xuefei Zhou and Yalei Zhang

State Key Laboratory of Pollution Control and Resources Reuse, Key Laboratory of Yangtze River Water Environment of MOE, College of Environmental Science and Engineering, Tongji University, Shanghai 200092, China
* Correspondence: jiangzhao@tongji.edu.cn (J.Z.); shenzheng@tongji.edu.cn (Z.S.); Tel.: 86-21-6598-5811

The supplementary Figures and Tables

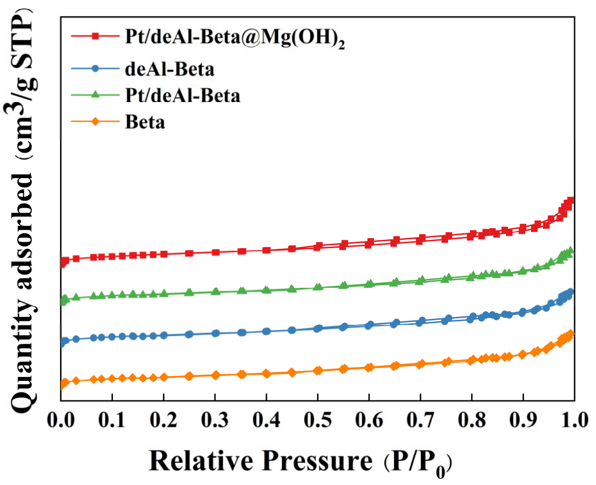

Figure S1. The Nitrogen adsorption-desorption isotherms of different catalysts.

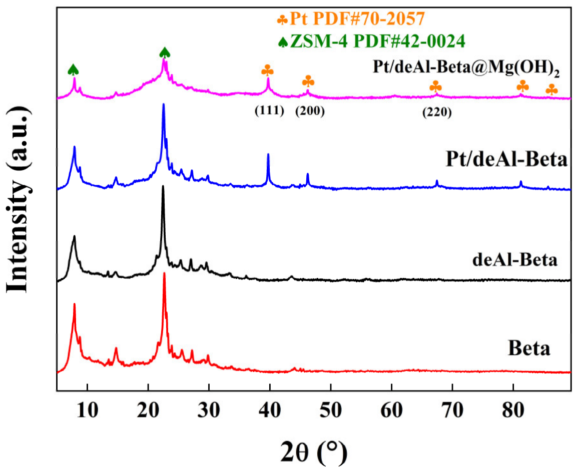

Figure S2. The XRD patterns of catalysts.

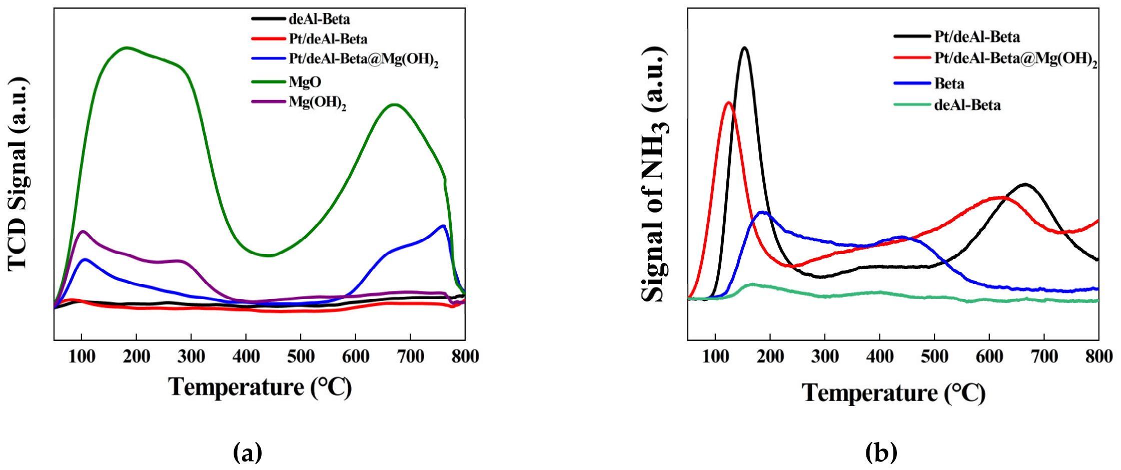

Figure S3. The results of the acid-base analysis. (a) CO_2-TPD results for different catalysts; (b) NH_3-TPD results for different catalysts.

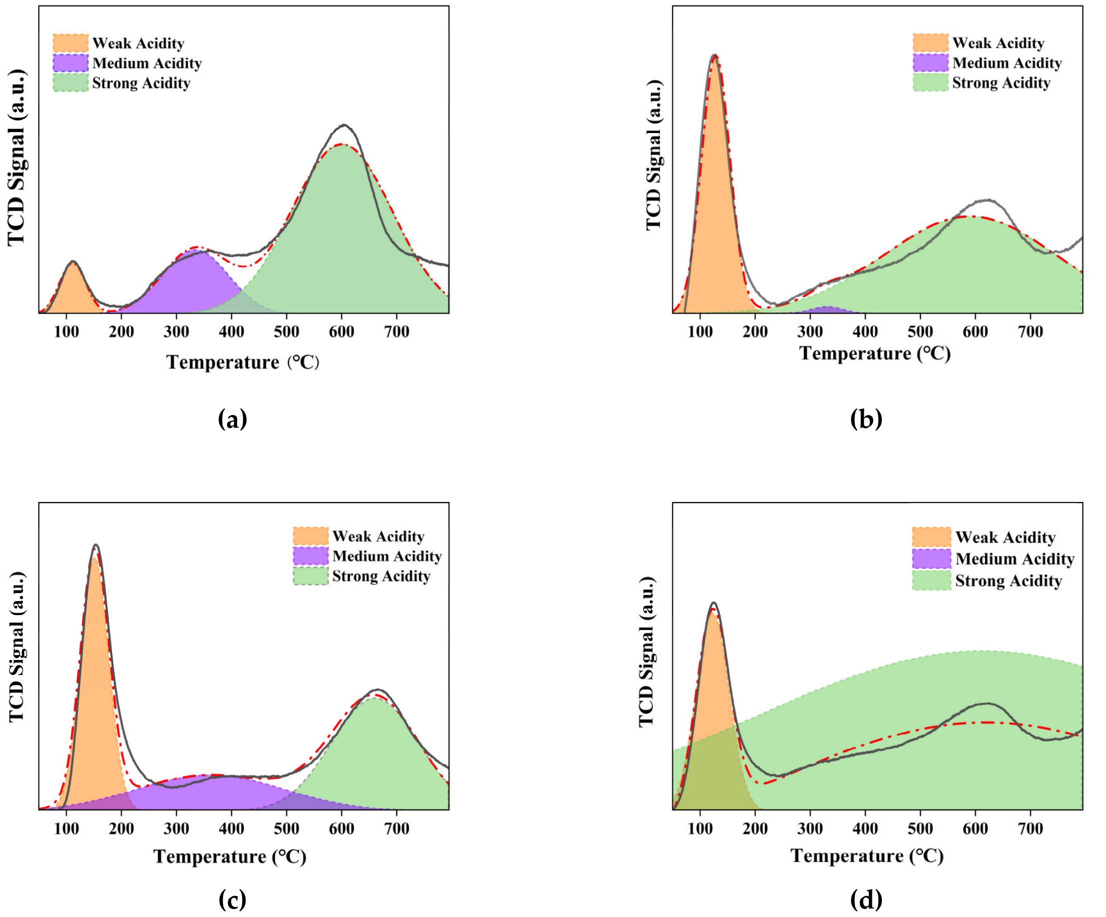

Figure S4. The results of the acid-base analysis. (a) CO_2-TPD results for Pt/deAl catalyst; (b) CO_2-TPD results for Pt/deAl@Mg(OH)$_2$ catalyst; (c) NH_3-TPD results for Pt/deAl catalyst; (d) NH_3-TPD results for Pt/deAl@Mg(OH)$_2$ catalyst.

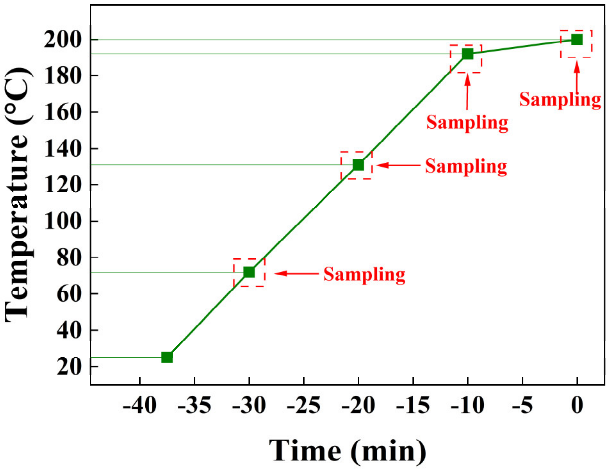

Figure S5. The temperature changes in the heating process before time 0.

Table S1. The pore properties of prepared catalysts.

Catalyst	Surface Area m^2/g	Pore Volume cm^3/g	Pore Size nm
Beta	428.19	0.36	3.40
deAl-Beta	411.69	0.37	3.64
Pt/deAl-Beta	301.75	0.33	4.42
Pt/deAl-Beta@$Mg(OH)_2$*	230.45	0.26	4.54

*The loading of $Mg(OH)_2$ is 7.5 wt%.

Table S2. The reaction performance of intermediate products over Pt/deAl-Beta and Pt/deAl-Beta@$Mg(OH)_2$.

Catalyst	Reactant	Conv. (%)	Selectivity(%) 1,2-PDO	EG*	Acetol	1,2-BDO*	1,2-HDO*	hexitol
Pt/deAl-Beta	glucose	100	4.3	0.8	21.2	7.7	3.8	6.8
Pt/deAl-Beta @$Mg(OH)_2$	glucose	100	34.1	8.5	0	4.3	2.5	3.1
Pt/deAl-Beta	fructose	100	35.4	6.2	5.9	3.6	5.2	2.8
Pt/deAl-Beta @$Mg(OH)_2$	fructose	100	38.6	4.8	0	1.9	3.3	5.3
Pt/deAl-Beta	sorbitol	2.7	0	0	0	0	81.7	-
Pt/deAl-Beta @$Mg(OH)_2$	sorbitol	3.3	58.6	0	0	0	0	-
Pt/deAl-Beta	mannitol	18.2	0	0	0	0	85.3	-
Pt/deAl-Beta @$Mg(OH)_2$	mannitol	39.1	68.7	0	0	0	6.7	-
Pt/deAl-Beta	1,2-HDO	7.6	0	0	0	0	-	-
Pt/deAl-Beta @$Mg(OH)_2$	1,2-HDO	9.7	0	0	0	0	-	-
Pt/deAl-Beta	DHA*	92.5	94.8	0	9.6	-	-	-
Pt/deAl-Beta @$Mg(OH)_2$	DHA	86.3	54.3	0	12.7	-	-	-
Pt/deAl-Beta	Acetol	90.2	81.3	0	-	-		-
Pt/deAl-Beta @$Mg(OH)_2$	Acetol	96.2	88.8	0	-	-		-

*1,2-PDO=1,2-propanediol; EG=ethylene glycol;.
1,2-HDO=1,2-hexanediol; 1,2-BDO=1,2-butanediol; DHA=dihydroxyacetone.

Table S3. Application of Pt loading in beta zeolite.

Catalyst	Pt loading(wt%)	Enhancing effect	References
Pt/H-Beta	0.5	guaiacol conversion larger than 90%	[1]
Pt/K-Beta-HS	1	longer lifetime(205 h), higher selectivity to aromatics (80%)	[2]
Pt/Beta	1	80% hydrogenation activity	[3]

Ni_2Pt/H-Beta	0.10	conversion of n-heptane is 55.9%, selectivity of isomerization is 91.5%	[4]
Pt/ZF-D	0.5	the highest catalytic performance of toluene combustion (T_{90} = 158 ℃)	[5]

References

1. Kim; Hannah; Jeon; Jong-Ki; Sang; Chai; Park; Sung; Hoon; Young-Kwon. Hydrodeoxygenation of guaiacol over Pt loaded zeolitic materials. *Journal of industrial and engineering chemistry* **2016**, *37*, 18-21.
2. B, Y.S.A.; B, Q.Z.A.; A, Z.Q.; A, Z.W.; A, W.J.; A, M.D.; A, W.F.; B, J.W.A. Hierarchically structured Pt/K-Beta zeolites for the catalytic conversion of n -heptane to aromatics. *Microporous and Mesoporous Materials* **2021**, *324.*
3. Monteiro, C.A.A.; Costa, D.; Zotin, J.L.; Cardoso, D. Effect of metal–acid site balance on hydroconversion of decalin over Pt/Beta zeolite bifunctional catalysts. *Fuel* **2015**, *160*, 71-79.
4. Yao, Y.; Tian, Y.-J.; Wu, H.-H.; Lu, S.-X. Pt-promoted and Hβ zeolite-supported Ni2P catalysts for hydroisomerisation of n-heptane. *Fuel Processing Technology* **2015**, *133*.
5. Zou, S.; Zhang, M.; Mo, S.; Cheng, H.; Fu, M.; Chen, P.; Chen, L.; Shi, W.; Ye, D. Catalytic Performance of Toluene Combustion over Pt Nanoparticles Supported on Pore-Modified Macro-Meso-Microporous Zeolite Foam. *Nanomaterials (Basel)* **2019**, *10*.
